# Association between Genetic Variants in *NOS2* and *TNF* Genes with Congenital Zika Syndrome and Severe Microcephaly

**DOI:** 10.3390/v13020325

**Published:** 2021-02-20

**Authors:** Julia A. Gomes, Eduarda Sgarioni, Juliano A. Boquett, Ana Cláudia P. Terças-Trettel, Juliana H. da Silva, Bethânia F. R. Ribeiro, Marcial F. Galera, Thalita M. de Oliveira, Maria Denise F. Carvalho de Andrade, Isabella F. Carvalho, Lavínia Schüler-Faccini, Fernanda S. L. Vianna

**Affiliations:** 1 Programa de Pós-Graduação em Genética e Biologia Molecular (PPGBM), Departamento de Genética, Universidade Federal do Rio Grande do Sul (UFRGS), Porto Alegre 91501-970, Brazil; eduarda.sgarioni@hotmail.com (E.S.); julianob9@hotmail.com (J.A.B.); lavinia.faccini@gmail.com (L.S.-F.); 2 Sistema Nacional de Informação sobre Agentes Teratogênicos (SIAT), Serviço de Genética Médica (SGM), Hospital de Clínicas de Porto Alegre (HCPA), Porto Alegre 90035-903, Brazil; 3 Instituto Nacional de Ciência e Tecnologia de Genética Médica Populacional (INAGEMP), Porto Alegre 90035-004, Brazil; 4 Laboratório de Medicina Genômica (LMG), Centro de Pesquisa Experimental (CPE), Hospital de Clínicas de Porto Alegre (HCPA), Porto Alegre 90035-903, Brazil; 5 Departamento de Enfermagem, Universidade do Estado de Mato Grosso (UNEMAT), Tangará da Serra 78300-000, Brazil; enfanacnp@gmail.com; 6 Secretaria Municipal de Saúde de Tangará da Serra, Tangará da Serra 78300-000, Brazil; epidemio@tangaradaserra.mt.gov.br; 7 Fundação Hospital de Clínicas do Acre (FUNDACRE), Rio Branco 69914-220, Brazil; bfrodrigues@gmail.com; 8 Departamento de Pediatria, Faculdade de Medicina, Universidade Federal de Mato Grosso (UFMT), Cuiabá 78048-902, Brazil; fgalera@uol.com.br; 9 Hospital Universitário Júlio Müller (HUJM), Universidade Federal de Mato Grosso (UFMT), Empresa Brasileira de Serviços Hospitalares (EBSERH), Cuiabá 78048-902, Brazil; thalitamara@yahoo.com.br; 10 Universidade Estadual do Ceará (UECE), Fortaleza 60741-000, Brazil; dra.denisecarvalho@gmail.com; 11 Centro Universitário Christus (UNICHRISTUS), Fortaleza 60811-020, Brazil; draisabellacarvalho@gmail.com; 12 Programa de Pós-Graduação em Ciências Médicas, Faculdade de Medicina, Universidade Federal do Rio Grande do Sul (UFRGS), Porto Alegre 91501-970, Brazil

**Keywords:** maternal exposure, Zika virus, Zika virus infection, teratogens, congenital abnormalities, genetic variation, genetic polymorphism, disease susceptibility, inflammation

## Abstract

Zika virus (ZIKV) causes Congenital Zika Syndrome (CZS) in individuals exposed prenatally. Here, we investigated polymorphisms in *VEGFA, PTGS2, NOS3, TNF*, and *NOS2* genes as risk factors to CZS. Forty children with CZS and forty-eight children who were in utero exposed to ZIKV infection, but born without congenital anomalies, were evaluated. Children with CZS were predominantly infected by ZIKV in the first trimester (*p* < 0.001) and had mothers with lower educational level (*p* < 0.001) and family income (*p* < 0.001). We found higher risk of CZS due the allele rs2297518[A] of *NOS2* (OR = 2.28, CI 95% 1.17–4.50, *p* = 0.015). T allele and TT/CT genotypes of the *TNF* rs1799724 and haplotypes associated with higher expression of *TNF* were more prevalent in children with CZS and severe microcephaly (*p* = 0.029, *p* = 0.041 and *p* = 0.030, respectively). Our findings showed higher risk of CZS due ZIKV infection in the first trimester and suggested that polymorphisms in *NOS2* and *TNF* genes affect the risk of CZS and severe microcephaly.

## 1. Introduction

Zika virus (ZIKV) is a human teratogen capable of causing neurological and ocular malformations in fetuses with in utero exposure to ZIKV infection [1,2]. Congenital Zika Syndrome (CZS) does not occur in all embryos or fetuses exposed, but in less than 42% [3,4]. The congenital anomalies present in children with CZS include brain calcifications, microcephaly, joint abnormalities, and ocular abnormalities, among others [4]. 

In order to understand the mechanisms associated with the development of congenital anomalies due to ZIKV infection, environmental and molecular variables have been investigated in humans, in vivo and in vitro; however, risk and protection factors still need to be better elucidated. The timing of ZIKV infection during pregnancy, for example, is a variable that has been discussed in studies as relevant to the occurrence and severity of congenital anomalies [5,6]. The investigation of molecular changes caused by ZIKV during infection, such as in the gene expression, is one approach used to understand its molecular mechanisms and factors associated with its teratogenesis. Studies in this context have shown an exacerbated activity of genes/proteins involved in the immune and inflammatory response during ZIKV infection [7,8,9,10]. 

It has been reported that some genes, such as *TNF, NOS2, PTGS2,* and *VEGFA*, as well as their proteins, are overexpressed during ZIKV infection, acting on the inflammatory response mechanism [11,12,13,14]. This neuroinflammatory profile in the central nervous system has been suggested as impairing the cell differentiation and proliferation—especially of neuroprogenitor cells—inducing the CZS [10,11,12]. The inefficient immune response as well as a highly inflammatory environment are, therefore, harmful scenarios for the developing brain, probably related to the increased cell death associated with the CZS development. Based on these data, the investigation of genes that act in this pathway and in developmental process, such as *VEGFA, PTGS2, NOS3, TNF,* and *NOS2*, as well as functional polymorphisms that affect the expression of these genes and their proteins activity may lead to the discovery of genetic factors of susceptibility to teratogenesis of ZIKV.

Hence, the aim of this study was to assess environmental variables, such as sociodemographic and clinical characteristics, as well as genetic variants in genes involved in the inflammatory process of response to ZIKV as risk or protective factors for CZS. Therefore, we investigated a sample of children who were in utero exposed to ZIKV infection and later developed CZS or, some of them, were born without congenital anomalies.

## 2. Materials and Methods 

### 2.1. Ethical Issues

This study was carried out following the rules of the Declaration of Helsinki and approved by the Ethics and Research Committee of the Hospital de Clínicas de Porto Alegre, the institution responsible for this study (nº 170619–CAAE 78735817910015327), and by all participating institutions. All legal guardians of individuals recruited for this study gave their informed consent for inclusion before they participated in the study.

### 2.2. Sample

In this case-control study, we included 40 children diagnosed with CZS whose mothers had evidence of ZIKV infection during pregnancy (case group) and 48 children without congenital anomalies whose mothers also had evidence of ZIKV infection (control group). Evidence of ZIKV exposure was defined as positive RT-PCR or specific symptoms of infection ZIKV during a ZIKV outbreak in the region during the pregnancy (e.g., rash, fever and/or joint pain). Case children were recruited between June 2018 to November 2019 from reports of microcephaly in five Brazilian research and/or assistance centers: North region (Fundação Hospital do Acre, *n* = 4), Northeast region (Fundação Universidade Estadual do Ceará, *n* = 21), Midwest region (Universidade do Estado de Mato Grosso, *n* = 2 and Universitário Júlio Muller, *n* = 12), and South region (Hospital de Clínicas de Porto Alegre, *n* = 1). Control children were recruited in the same research and/or assistance centers from the North region (*n* = 1), Midwest (*n* = 46, from a cohort of women that gave birth in 2016, in the city of Tangará da Serra), and South region (*n* = 1). 

Children with CZS included in this study were, in the first year of life, attended by a team of doctors, including geneticists, neuropediatricians, neurologists, ophthalmologists, physiotherapists, and (or) dentists. Subsequently, according to the needs related to the neurodevelopment of each child, they continued to be accompanied by some professionals, including pediatricians, physiotherapists, ophthalmologists, dentists, and (or) neurologists, among others, who are part of this study. Therefore, the clinical characteristics of these individuals, as well as ophthalmological and neuroimaging tests were obtained, when available, through the medical records of these consultations or through questionnaires applied to the mothers. Likewise, sociodemographic data were obtained through questionnaires applied during the medical consultation.

### 2.3. Genetic Analysis

A blood or saliva sample was collected from individuals and DNA extraction was performed by the FlexiGene DNA Kit (Qiagen, Hilden, Germany) or Oragene Kit (DNA Genotek, Ottawa, Ontario, Canada). Eleven polymorphisms in *VEGFA, PTGS2, NOS3, TNF,* and *NOS2* genes were selected to be evaluated in this study. Information about the polymorphisms evaluated and the TaqMan assays used are found in Appendix A. The criteria for genetic variants selection were based on: (1) Minor Allele Frequency (MAF) > 1% (based on gnomAD database information for non-Finnish European and/or AbraOM database for Brazilian), and (2) functional description as modifiers of their gene’s expression or protein function. The genotyping was performed through the TaqMan Genotyping Assay method in Step One PlusTM Real-Time PCR Systems (Thermo Fisher Scientific, Waltham, Massachusetts, EUA). 

### 2.4. Statistical Analyses 

A descriptive analysis of the congenital anomalies of individuals with CZS was performed. Quantitative variables were tested through the Shapiro–Wilk test to verify their normality and according to the distribution found, Student’s *t* test or Mann–Whitney *U* test were applied. The Hardy–Weinberg equilibrium was tested for all polymorphisms. Categorical variables were compared between the groups by Chi-square test or Fisher’s Exact Test. Through a univariate logistic regression analysis, we looked for associations between individual variables and the occurrence of CZS. *p*-values lower than 0.05 were considered to be significant. The SPSS v.18 software was used to perform the statistical analyses (IBM, Armonk, New York, NY, USA).

Linkage disequilibrium (LD) analysis was inferred with Haploview v.4.2. software (Broad Institute, Cambridge, MA, USA). The haplotypes were obtained with the Bayesian algorithm in Phase 2.1.1 software.

## 3. Results

### 3.1. Sociodemographic and Clinical Profile of Children Who Were In Utero Exposed to ZIKV Infection

Eighty-eight children who were in utero exposed to ZIKV infection were recruited for this study. Forty children developed Congenital Zika Syndrome (CZS) (case group) and forty-eight were born without alterations (control group). The sociodemographic characteristics of the children are presented in Table 1. There was a higher prevalence of black individuals in both case (77%) and control (65%) groups and, therefore, this variable did not present a statistically significant difference between the groups. As expected, weight, height, and the cephalic perimeter were higher in the control group (*p* < 0.001). Most mothers of children with CZS were exposed to ZIKV infection in the first trimester (80%) while most mothers of children without CZS were exposed to ZIKV infection the third trimester (44%) (*p* < 0.001). Cesarean section had a significantly higher prevalence in the control group (90%) (*p* < 0.001). Mothers of children with CZS had a lower educational (*p* < 0.001) and lower monthly family income (*p* = 0.008).

**Figure 1:**
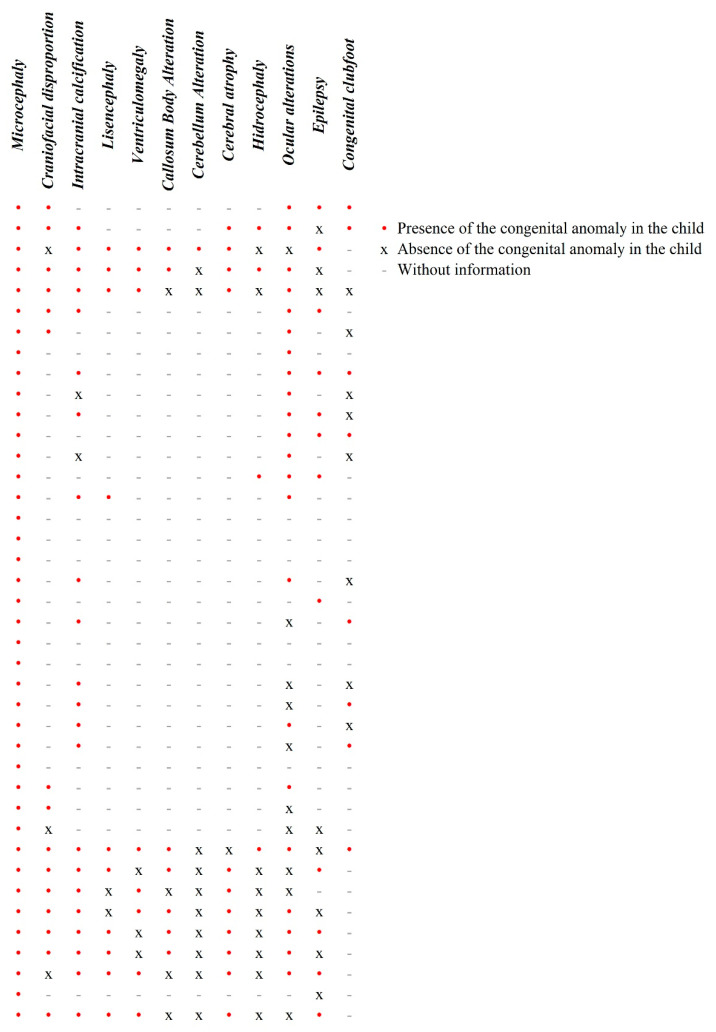
Descriptive analysis of the clinical findings of each individual of the case group.

Congenital anomalies of the children with CZS are presented in Figure 1. Such anomalies were identified during the children’s first year of life by a team of doctors and through dysmorphological, neuroimaging, and ophthalmic examinations that are available in some of the research and assistance centers in which the children have been treated.

For those whom the exams were performed and the information was available, the most prevalent clinical findings were microcephaly (*n* = 40–100%), calcifications (*n* = 22/24–92%), cerebral atrophy (*n* = 11/12–92%), lissencephaly (*n* = 10/12–83%), craniofacial disproportion (*n* = 15/18–83%), ventriculomegaly (*n* = 8/11–73%), and ocular alterations (*n* = 22/32–69%).

Given the neurological impairment of most of these children, they continued to be accompanied by specific doctors, such as pediatricians and physiotherapists (more regularly), or ophthalmologists, dentists, and neurologists (more sporadically). Such monitoring varied again depending on the research and assistance center in which the children have been treated.

### 3.2. Genetic Susceptibility to CZS and Severe Microcephaly

The allelic and genotypic frequencies of polymorphisms in the *VEGFA, PTGS2, NOS3, TNF,* and *NOS2* genes in both groups are presented in Table 2. The genotypic frequencies of the rs1799983 (*NOS3*), rs1799724 (*TNF*), rs2779249, and rs2297518 (*NOS2*) in the control group were not consistent with the Hardy–Weinberg equilibrium as well as the genotypic frequencies of the rs2297518 (*NOS2*) in the case group. 

The genotypic frequencies of the rs1799983 (*NOS3*) were different between the groups (*p* = 0.045). Moreover, there was a higher frequency of the A allele of the rs2297518 (*NOS2*) in the case group (*p* = 0.019). The allelic and genotypic frequencies of the other polymorphisms showed no statistically significant difference between the groups.

The frequencies of haplotypes generated by combining alleles of variants in the same gene were also compared between groups (Table 3). The frequencies of the *VEGFA* haplotypes were statistically different between cases and controls (*p* = 0.002). Although, *NOS2* haplotypes also presented a differential frequency between the groups, these were not statistically significant (*p* = 0.054). The haplotypic frequencies of the other genes showed no statistically significant difference between the groups. In the *TNF* gene, the TCG haplotype (rs1799964, rs1799724, and rs361525, respectively) contained risk alleles associated with low *TNF* expression [15,16,17,18]. The frequency of this haplotype was compared between cases and controls, but there was no statistically significant difference.

Table 4 presents the results of a univariate logistic regression analysis of significant risk variables for the CZS. The first trimester of exposure to the ZIKV infection was related to a higher risk to CZS development (OR: 19.38, CI 95% 4.70–133.78, *p* < 0.001). Furthermore, the presence of the A allele of the rs2297518 (*NOS2*) was related to a higher risk to the CZS (OR: 2.28, CI 95% 1.17–4.50, *p* = 0.015).

A specific phenotype of the CZS, the severity of the microcephaly, was evaluated in the case group. Mild or moderate microcephaly was defined if the head circumference was between 2 and 3 standard deviation (SD) below the mean for sex and age, and severe if it was 3 or more SD below the mean [19]. Children with 2SD of microcephaly were compared to children with 3SD of microcephaly for the frequency of two variables already reported in the literature as related to these phenotypes—trimester of ZIKV infection and *TNF* gene [20,21]. The results of such analysis are presented in Table 5. The frequency of ZIKV infection in the first trimester was higher in children with severe microcephaly (100%), compared with children with moderate microcephaly (33%) (*p* = 0.019). Children with severe microcephaly presented higher frequency of the T allele and genotypes TT or CT of the *TNF* rs1799724, compared with children with moderate microcephaly (*p* = 0.029 and *p* = 0.041, respectively). Moreover, the two groups presented a differential frequency of *TNF* haplotypes (*p* = 0.030), with an especially higher frequency of the TCG haplotype, which contains all the alleles related to the lower expression of *TNF* [15,16,17,18]. This haplotype is found in 88% of the children with moderate microcephaly (<2SD) against 58% in children with severe microcephaly (<3SD) (*p* = 0.079).

## 4. Discussion

Zika virus teratogenic potential has been discovered in the recent years; thus, little is known about the susceptibility factors and mechanisms related to the adverse effects caused by its exposure in embryos and fetuses in development so far. It is known that around 1 to 42% of embryos or fetuses with in utero exposure to ZIKV infection developed the CZS [4]. Discordant twins for CZS have been shown to be not uncommon [9]. Based on this, it is important to take into account the environmental and genetic factors that may confer susceptibility to ZIKV teratogenesis. In this work, we evaluated some sociodemographic, clinical, and genetic variants as possible risk factors to CZS in a sample of children who were in utero exposed to ZIKV infection, comparing children who were born with and without CZS.

Regarding some gestational risk factor to CZS, the exposure to ZIKV in the first trimester of pregnancy has been reported as a risk factor to CZS, presenting more severe congenital anomalies than later exposures [2,6]. Our results corroborate these findings, since we found a higher prevalence of children with CZS who were exposed in utero to ZIKV in the first trimester of pregnancy while children born without congenital anomalies were predominantly exposed in second and third trimester of gestation. Moreover, we found a higher frequency of the first trimester exposure in children with severe microcephaly.

Still, in this context of environmental variables possibly involved with the teratogenic effects of ZIKV, the socioeconomic level has been discussed as a possible environmental factor associated with this asymmetric distribution of CZS in Brazil [22]. That discussion is important, since this variable was also associated with the availability and quality of mothers’ diets during pregnancy, a condition recently also associated with the development of CZS [23]. In the present sample, we observed that families of children with CZS had a lower socioeconomic level compared to families of children without CZS. Mothers in the case group had a lower educational level and reported lower family income. This finding this is not new in the literature, since low socioeconomic level has been described as a risk factor for congenital anomalies. Therefore, it is probably that this population presents a higher risk for negative outcomes after ZIKV infection during pregnancy [24,25].

In relation to genetic risk factors, through the analyses of functional genetic variants in *VEGFA, PTGS2, NOS3, TNF*, and *NOS2*, genes related to immune and inflammatory response, we found different allelic, genotypic, and haplotypic frequencies between children with and without CZS. Regarding the *NOS2* gene, we found a higher prevalence of the rs2297518 allele A in children with CZS, being that this allele is associated with an increased risk to CZS (OR: 2.28 (95% IC 1.17–4.50). *NOS2* rs2297518 is a functional polymorphism that affects the iNOS protein activity, with the A allele related to increased protein activity and higher nitric oxide (NO) production [26,27]. NO has been reported as playing major roles in neurogenesis and neurodevelopment, and its dosage is extremely important [28,29]. The dysregulation of NO has been involved in the progression of many neurodevelopmental, neurobehavioral, and neurodegenerative disorders [29]. In this sense, the dysregulation of NO during ZIKV infection, caused both by the host response to the virus and by the individual’s genotype, could affect the development process causing a congenital anomaly.

Similarly, the haplotypic frequencies of two genes, *VEGFA* and *NOS2*, were different between children with and without CZS. The comparison of haplotype frequencies between cases and controls has been suggested as helping to identify overlapping haplotypes among affected individuals, representing a shared region that contains a genetic risk factor [30]. We highlight that the *NOS2* gene haplotypic frequency was different between the two groups of children who were in utero exposed to ZIKV infection; however, it has not reached statistical significance, probably due to the sample size of the groups. On the other hand, *VEGFA* presented a statistically significant difference of its haplotype frequencies between the two groups. *VEGFA* is an important gene during neurodevelopment, acting on the neurogenesis, neuronal differentiation, and angiogenesis processes [31,32]. Genetic variants and haplotypes that affect the expression of this gene have the potential to impact and impair these developmental processes [31]. In the context of ZIKV infection during the developing brain, where the expression of this gene seems to be already affected, the presence of genetic variants and haplotypes that decreased its expression could modulate the impact of ZIKV for a worse scenario, further damaging neurogenesis and processing angiogenesis.

Focusing on a specific phenotype of the CZS, the severity of the microcephaly, we found an association of it and the *TNF* gene. The frequency of the rs1799724[T] was higher in children with a severe microcephaly. This allele has been related to the higher expression of the *TNF* gene [15,16]. The haplotypes of *TNF* also showed a different frequency between children with CZS and moderate or severe microcephaly, but in this scenario, a higher frequency of the TCG haplotype was found in children with a moderate microcephaly. This haplotype is composed by the three alleles of the variants rs1799964[T], rs1799724[C], and rs361525[G] of *TNF* related to the low expression of the gene [15,16,17,18]. TNF is a cytokine that, among many functions, has pro-inflammatory effects during a viral infection [33]. The immunological and inflammatory effects caused by TNF act in the eradication of infectious agents, but they can cause tissue damage, cell death, and systemic effects [33]. In this sense, the higher frequency of the rs1799724[T] in children with severe microcephaly, leading to a greater expression of *TNF*, combined with the natural increase of *TNF* expression to combat the ZIKV infection could be associated to this most severe phenotype in these individuals due to an exacerbated immunological and inflammatory response in the neural cells. On the other hand, the higher frequency of the TCG haplotype of *TNF* in individuals with moderate microcephaly, which is associated with the lower expression of *TNF*, could also explain this less severe phenotype in these individuals.

It is important to highlight that the polymorphisms evaluated in this study affect the expression of their genes or function of the proteins, which act on the fetus’ brain, both in the context of development and inflammatory response [34,35,36,37,38,39]. Since the aim of this study was evaluating the role that these polymorphisms could have, in the context of the ZIKV infection, on the fetal brain development, we considered that it would be interesting to evaluate their frequency only in individuals with CZS and individuals who have been exposed in utero to the ZIKV infection but were born normal and, in this sense, the parents were not evaluated, since this analysis would not add information to answer to the aim of the study. 

In addition, although these genes and polymorphisms have an important role in neurogenesis or neuroinflammation, they have not been described as capable of causing the phenotypes seen in CZS, such as microcephaly, among others outside the context of the ZIKV infection [34,35,36,37,38,39,40,41]. This could mean that ZIKV infection potentiates the effect of these polymorphisms and, for this reason, they act on individuals’ susceptibility to CZS in the context of ZIKV infection. 

Despite the interesting results of the present study, some limitations of should be considered for the results to be interpreted in a clear and unbiased manner. Firstly, the sample size of our sample is a limiting factor in the study, as it restricts our power to make strong associations and prevents us from performing more robust statistical analyses. Moreover, it is important to highlight that the number of children with CZS coming from a given region was not matched with the number of children without CZS coming from the same region. These two aspects of the sample may have been responsible for the fact that some genotypic frequencies found in this work were not consistent with the Hardy–Weinberg equilibrium. In this sense, the associations observed in the present study need to be confirmed by other studies in order better understand their real impact on CZS. Although the sample was obtained in partnership with several Brazilian institutions, the recruitment of new and well documented cases is difficult, taking into account that the ZIKV outbreak in Brazil has decreased considerably since 2017. In addition, there are a great number of cases where the confirmation of infection has been lost and, therefore, they were not included in this study. Regarding the clinical description of the individuals in the case group, the data were not available to all individuals due to the resources of the institution where they were attended and their possibility of carrying out exams.

The investigation for risk factors for the ZIKV teratogenesis and for the understanding of its molecular mechanisms still has much to advance, since little is known about this. Teratogenesis is a complex event and, therefore, there is not only one factor that can explain the susceptibility to damage caused by a teratogen exposure. In this study, we reported an interesting association of alleles and haplotypes of *NOS2, VEGFA,* and *TNF* genes with the CZS development and severity of the microcephaly in the CZS. Moreover, our results corroborate that the exposure to ZIKV in the first trimester of pregnancy is associated with the CZS as well as with the severity of the microcephaly in the affected individuals. Similarly, we found the socioeconomic level as a possible environmental risk factor to CZS. Future studies must explore such variables in larger samples as well as explore other possible risk factors to the ZIKV teratogenesis.

## Figures and Tables

**Table 1:** Evaluation of Clinical, Gestational, and Sociodemographic Characteristics in the Case and Control Groups.

Variables	Case † (*n* = 40)	Control (*n* = 48)	*p*-Value ‡
Sex (n, %)			
*Male*	23 (57%)	26 (54%)	0.754
*Female*	17 (43%)	22 (46%)	
Ethnicity (n, %)			
*Black*	31 (77%)	31 (65%)	0.242
*White*	9 (23%)	17 (35%)	
Weight (kg)	2.5 (2.2–2.9)	3.2 (2.8–3.5)	<0.001 *
Height (cm)	45.0 (44.0–48.0)	49.0 (47.0–50.0)	0.001 *
Cephalic perimeter (cm)	29.0 (27.3–31.0)	35.0 (34.0–36.0)	<0.001 *
Gestational age at birth (weeks)	38.0 (37.0–39.0)	38.0 (37.0–38.7)	0.522
Types of delivery (n, %)			
*Vaginal delivery*	16/30 (53%)	5 (10%)	<0.001 *
*Cesarean section*	14/30 (47%)	43 (90%)	
Mother’s age (years)	28.0 (22.5–35.5)	29.5 (22.0–33.0)	0.824
Father’s age (years)	28.0 (24.0–37.8)	31.0 (26.0–35.0)	0.693
Trimester of ZIKV infection (n, %)			
*1st*	24/30 (80%)	13 (27%)	<0.001 *
*2nd*	4/30 (13%)	14 (29%)	
*3rd*	2/30 (7%)	21 (44%)	
Exposure during pregnancy (n, %)			
*Alcohol*	3/35 (9%)	14 (29%)	0.028 *
*Smoke*	0/35	1 (2%)	1.000
*Drugs*	0/35	1 (2%)	1.000
Maternal yellow fever vaccine (n, %)	18/24 (75%)	35 (73%)	1.000
Maternal educational level (n, %)			
*Elementary school*	13/38 (34%)	0	<0.001 *
*High school*	13/38 (34%)	2 (4%)	
*Incomplete or complete higher education*	12/38 (32%)	46 (96%)	
Monthly family income (n, %)			
*Less than 3 minimum wages*	23/27 (85%)	12/25 (46%)	0.008 *
*Between 3 and 9 minimum wages*	4/27 (15%)	12/25 (46%)	
*More than 9 minimum wages*	0	2/25 (8%)	

† In the case group, some information was not available or was not answered for all mothers. ‡ Quantitative variables were compared between the groups through the Student’s *t* test or Mann–Whitney *U* test and categorical variables through the Chi-squared test or Fisher’s exact test; Quantitative variables are presented as median and quartiles; * Statistically significant.

**Table 2:** Allelic and genotypic frequencies of polymorphisms in *VEGFA, PTGS2, NOS3, TNF,* and *NOS2* genes in the case and control groups.

Gene	Polymorphism	Allele/Genotype	Case (*n* = 40)	Control (*n* = 48)	*p*-Value †
***VEGFA* (n, %)**	rs1570360	G	70 (87%)	72 (75%)	0.054
		A	10 (13%)	24 (25%)	
		GG	30 (75%)	28 (58%)	0.109
		GA	10 (25%)	16 (33%)	
		AA	0	4 (9%)	
	rs2010963	G	39 (49%)	47 (49%)	1.000
		C	41 (51%)	49 (51%)	
		GG	11 (27%)	11 (23%)	0.821
		GC	18 (46%)	25 (52%)	
		CC	11 (27%)	12 (25%)	
	rs3025039	C	61 (76%)	84 (87%)	0.073
		T	19 (24%)	12 (13%)	
		CC	25 (63%)	36 (75%)	0.084
		CT	11 (27%)	12 (25%)	
		TT	4 (10%)	0	
***PTGS2* (n, %)**	rs689465	T	68 (85%)	77 (80%)	0.434
		C	12 (15%)	19 (20%)	
		TT	29 (73%)	30 (63%)	0.746
		TC	10 (25%)	17 (35%)	
		CC	1 (2%)	1 (2%)	
***NOS3* (n, %)**	rs2070744	T	57 (71%)	63 (66%)	0.516
		C	23 (29%)	33 (34%)	
		TT	20 (50%)	21 (44%)	0.679
		TC	17 (42%)	21 (44%)	
		CC	3 (8%)	6 (12%)	
	rs1799983	G	65 (81%)	73 (76%)	0.464
		T	15 (19%)	23 (24%)	
		GG	27 (67%)	25 (52%)	0.045 *
		TG	11 (28%)	23 (48%)	
		TT	2 (5%)	0	
***TNF* (n, %)**	rs1799724	C	70 (87%)	86 (90%)	0.812
		T	10 (13%)	10 (10%)	
		CC	31 (78%)	40 (83%)	0.606
		CT	8 (20%)	6 (13%)	
		TT	1 (2%)	2 (4%)	
	rs361525	G	76 (95%)	93 (97%)	0.703
		A	4 (5%)	3 (3%)	
		GG	36 (90%)	45 (94%)	0.694
		GA	4 (10%)	3 (6%)	
	rs1799964	T	63 (79%)	75 (80%)	1.000
		C	17 (21%)	19 (20%)	
		TT	25 (62%)	31 (66%)	0.939
		TC	13 (33%)	13 (28%)	
		CC	2 (5%)	3 (6%)	
***NOS2* (n, %)**	rs2779249	C	28 (35%)	40 (42%)	0.437
		A	52 (65%)	56 (58%)	
		CC	6 (15%)	12 (25%)	0.521
		CA	16 (40%)	16 (33%)	
		AA	18 (45%)	20 (42%)	
	rs2297518	G	50 (62%)	76 (79%)	0.019 *
		A	30 (38%)	20 (21%)	
		GG	20 (50%)	33 (69%)	0.144
		GA	10 (25%)	10 (21%)	
		AA	10 (25%)	5 (10%)	

† Chi-squared test or Fisher’s exact test; * Statistically significant.

**Table 3:** Frequencies of the haplotypes generated by the combination of alleles of the polymorphisms in *VEGFA, NOS3, TNF,* and *NOS2* genes in case and control groups.

Gene	Haplotypes †	Cases (*n* = 40)	Controls (*n* = 48)	*p*-Value §
***VEGFA* (n, %)**	**GGC**	30 (38%)	23 (24%)	0.002 *
	**GCC**	25 (31%)	40 (42%)	
	**GCT**	14 (17%)	9 (9%)	
	**AGC**	4 (5%)	21 (22%)	
	**AGT**	4 (5%)	3 (3%)	
	**ACC**	2 (3%)	0	
	**GGT**	1 (1%)	0	
***NOS3* (n, %)**	**TG**	55 (69%)	55 (57%)	0.156
	**CT**	14 (17%)	15 (16%)	
	**CG**	9 (11%)	18 (19%)	
	**TT**	2 (3%)	8 (8%)	
***TNF* (n, %)**	**TCG**	54 (66%)	68 (71%)	0.831
	**CCG**	14 (16%)	16 (17%)	
	**TTG**	10 (13%)	9 (9%)	
	**CCA**	4 (5%)	3 (3%)	
	**TCG** ‡	53 (70%)	68 (73%)	0.732
	**others** ‡	23 (30%)	25 (27%)	
***NOS2* (n, %)**	**GA**	37 (46%)	51 (53%)	0.054
	**AA**	15 (19%)	7 (7%)	
	**AC**	15 (19%)	13 (14%)	
	**GC**	13 (16%)	25 (26%)	

† *VEGFA* haplotypes: variants rs1570360, rs2010963, and rs3025039, respectively; *NOS3:* rs2070744 and rs1799983, respectively; *TNF:* rs1799964, rs1799724, and rs361525, respectively; *NOS2:* rs2297518, and rs2779249, respectively; ‡ Comparison of the frequencies of a haplotype containing all alleles associated with low expression of the *TNF* against the other haplotypes; § Chi-squared test or Fisher’s exact test; * Statistically significant.

**Table 4:** Univariate logistic regression showing risk variables for the Congenital Zika Syndrome.

Risk Variables	Odds Ratio (95% IC)	*p*-Value
1st trimester of ZIKV infection	19.38 (95% IC 4.70–133.78)	<0.001 *
*NOS2* rs2297518[A]	2.28 (95% IC 1.17–4.50)	0.015 *

[A]: allele A of the *NOS2* rs2297518 genetic variant; * Statistically significant.

**Table 5:** Association between trimester of Zika virus (ZIKV) infection during pregnancy or *TNF* polymorphisms and the severity of microcephaly in cases with CZS.

Variables	Allele/ Genotype/ Haplotype	Severity of Microcephaly		
		Mild (*n* = 8)	Severe (*n* = 12)	*p*-Value
**Trimester of ZIKV infection** † **(n, %)**				
*1st*		2 (33%)	7 (100%)	0.019
*2nd*		3 (50%)	0	
*3rd*		1 (17%)	0	
**TNF (n, %)**				
rs1799724	T	0	7 (29%)	0.029 *
	C	16 (100%)	17 (71%)	
	CC	8 (100%)	6 (50%)	0.041 *
	CT	0	5 (42%)	
	TT	0	1 (8%)	
rs361525	A	1 (6%)	0	0.400
	G	15 (94%)	24 (100%)	
	GG	7 (87%)	12 (100%)	0.400
	AG	1 (13%)	0	
rs1799964	C	2 (13%)	3 (13%)	1.000
	T	14 (87%)	21 (87%)	
	TT	6 (75%)	9 (75%)	1.000
	CT	2 (25%)	3 (25%)	
Haplotypes ‡	CCG	1 (6%)	3 (13%)	0.030 *
	CCA	1 (6%)	0	
	TCG	14 (88%)	14 (58%)	
	TTG	0	7 (29%)	
	TCG ^§^	14	14	0.079
	others ^§^	2	10	

† The information about the trimester of exposure to ZIKV infection during pregnancy was not available for all children with data of the severity of the microcephaly; ‡ *TNF* haplotypes: variants rs1799964, rs1799724, and rs361525, respectively; § Comparison of the frequencies of a haplotype containing all the alleles associated with low expression of the *TNF* against the other haplotypes; Chi-squared test or Fisher’s exact test; * Statistically significant.

## Data Availability

The data presented in this study are available on request from the corresponding author. The data are not publicly available due to privacy.

## Appendix A

**Table A1:** General information of the polymorphisms evaluated.

Gene	Polymorphism	Commercial Assay Codes †	Allelic Frequency ‡	Allelic Frequency §	Gene Location	Impact on the Gene or Protein
***VEGFA***	rs1570360	C_1647379_10	A = 34%	A = 24%	promoter	*VEGFA* gene expression
	rs2010963	C_8311614_10	C = 29%	C = 36%	5′UTR	*VEGFA* gene expression
	rs3025039	C_16198794_10	T = 15%	T = 13%	3′UTR	*VEGFA* mRNA concentration
***PTGS2***	rs689465	C_2517146_10	C = 12%	-	promoter	In combination with other genetic variants in haplotypes, it affects *PTGS2* expression
***NOS3***	rs2070744	C_15903863_10	C = 37%	-	intron	*NOS3* gene expression
	rs1799983	C_3219460_20	T = 32%	T = 29%	missense	eNOS enzyme activity
***TNF***	rs1799724	C_11918223_10	T = 9%	-	promoter	*TNF* gene expression
	rs361525	C_2215707_10	A = 4%	A = 5%	promoter	*TNF* gene expression
	rs1799964	C_7514871_10	C = 20%	C = 24%	promoter	*TNF* gene expression
***NOS2***	rs2779249	C_2593689_10	A = 29%	-	intron	iNOS protein activity
	rs2297518	C_11889257_10	A = 19%	A = 17%	missense	iNOS protein activity

† TaqMan assays used to genotype the polymorsphisms in this study; ‡ Data from gnomAD database (European non-Finnish population); § Data from AbraOM database (Brazilian population).
